# Distinct Gut Microbiota Structure and Function of Children with Idiopathic Central and Peripheral Precocious Puberty

**DOI:** 10.1155/2022/7175250

**Published:** 2022-11-09

**Authors:** Congfu Huang, Haiying Liu, Wei Yang, Yinhu Li, Bin Wu, Junru Chen, Zhenyu Yang, Cuifang Liao, Limei Liu, Xiaowei Zhang

**Affiliations:** ^1^Department of Pediatrics, Longgang District Maternity & Child Healthcare Hospital, Shenzhen, China; ^2^Department of Pediatrics, Affiliated Shenzhen Maternity and Child Healthcare Hospital, Southern Medical University, Shenzhen, China; ^3^Department of Pediatrics, The People's Hospital of Shenzhen Baoan District, Shenzhen, China; ^4^School of Statistics and Data Science, NanKai University, Tianjin, China; ^5^Department of Computer Science, City University of Hong Kong, Hong Kong, China; ^6^Department of Obstetrics and Gynaecology, Peking University Shenzhen Hospital, Shenzhen, Guangdong, China

## Abstract

Precocious puberty (PP) is one of the most common endocrine diseases in children, and the pathogenesis is currently unknown. Recent studies on the gut-brain axis have shown that there is a correlation between childhood endocrine diseases and the gut microbiota (GM). To explore the GM characteristics of children with different types of PP, we recruited 27 idiopathic central precocious puberty children (ICPP group), 18 peripheral precocious puberty children (PPP group), and 23 healthy children of the same age (HC group). Their stool samples were subjected to 16S rDNA sequencing. In this study, we found that the OTUs numbers, the annotated genera, and *α*-diversity of GM of the ICPP and PPP group were all significantly higher than that in the HC group (*P* < 0.05). The abundance of butyrate-producing bacteria *Prevotella*, *Lachnospiracea incertae sedis*, *Roseburia*, *Ruminococcus,* and *Alistipes* was significantly higher in the ICPP group and the PPP group, and *Bacteroides* and *Faecalibacterium* showed significantly higher abundance in the HC group. The GM symbiosis network showed that both *Bacteroides* and *Faecalibacterium* were negatively correlated with these butyrate-producing bacteria. The abundances of most significantly changed genera were gradually increased from HC to PPP, and to the ICPP group, while only Bacteroides was gradually decreased. After the prediction of the metabolic pathways of the GM, the cell motility, signal transduction, and environmental adaptation were significantly enriched in the ICPP and the PPP groups (*P* < 0.05), while the carbohydrate metabolism pathway was significantly lower (*P* < 0.001). Overall, this study showed that the GM composition and predicted functional pattern of children with ICPP and PPP are different from healthy children, and PPP may be a transitional stage between ICPP and HC children, which provide a theoretical basis for clinical intervention based on GM in the treatment of PP.

## 1. Introduction

Precocious puberty (PP) refers to the development of secondary sexual characteristics when a children's puberty developed before the age of 8, and it affects children's growth, development, social and psychological health [1]. In China, the incidence of PP of urban children is 4%–7%, which is 2–5 percentage points higher than that of rural children [2]. According to the early start of hypothalamic-pituitary-gonadal axis (HPGA) function, PP can be divided into central precocious puberty (CPP), peripheral precocious puberty (PPP), and not complete precocious puberty (also called partial precocious puberty). Among them, the incidence of CPP is remarkably high, which is reached about 1/5000∼1/10000. While, among all the CPP cases, girls are about 5∼10 times higher than that of boys [3], and 80∼90% of the girls are diagnosed as idiopathic central precocious puberty (ICPP), the latter refers to CPP caused by noncentral nervous system (CNS) lesions.

PP is closely related to children's nutritional status and body fat mass, which are results from the dietary habits and structure [4, 5]. According to the previous studies, the metabolites profile of the blood and urine samples from CPP children and healthy children with same ages was significantly different, for example, the concentration of homovanillic acid and vanillylmandelic acid (the major end products of catecholamine metabolism) in the urine of CPP children increased [6, 7]; the blood production of prostaglandin E2, prostaglandin F1*α*, prostaglandin F2*α*, leukotriene D4, 5-hydroxydocosatetraenoic acid, and 5-hydroxyeicosatetraene acid (related to the arachidonic acid metabolic pathway) increased [8].

In recent years, increasing evidences have provided to confirm that gut microbiota (GM) as another organ of the human body plays a crucial role in many metabolic diseases, especially for the diseases closely related to nutritional metabolites, such as obesity, hyperlipidemia, and diabetes [9–11]. Under the consideration of the common association between obesity and PP, the relationship between PP and GM has also been explained, and distinct microbiota structure was found in PP cases [10, 12]. Dong et al. found that intestinal-enriched bacteria in ICPP girls are related to the production of short-chain fatty acids (SCFAs) and obesity [13]. In addition, the imbalance of GM could lead to the alteration of nitric oxide (NO) synthesis and gut-brain axis (GBA) activation, which also contributes to the connection between CPP and obesity [14]. However, there were several clinical subgroups of the PP cases, and studies have revealed that some children with PPP can be converted to CPP [15]. However, whether there is a correlation between children's GM with different types of PP remains unclear.

In the current study, we recruited 18 PPP children, 27 ICPP children, and 23 healthy children (HC) to study the GM characteristics of different types of PP. In addition to explore the GM differences among the different types of PP, we also assessed the correlation of GM between the three groups, and predicted the alter of GM function and their corresponding symbiosis networks. We hope that this study can deepen our understanding of the role of GM in the pathogenesis of PP and provide a theoretical basis for PP children's clinical intervention based on GM.

## 2. Materials and Methods

### 2.1. Participant Recruitment

PPP and ICPP patients were recruited by the Longgang District Maternity and Child Health Hospital, Shenzhen, China, and confirmed by the Children's Health Department of the hospital. The participants were all girls, aged between 6 and 10 years old. The diagnostic criteria for PPP referred to the “A pediatrician's guide to central precocious puberty” [16] as follows: (I) the early appearance of secondary sexual characteristics (before 8 years old); (II) the abnormal developmental procedures of the sex signs; (III) the size of the gonads is at the prepubertal level; (IV) the gonadotropin is at the prepubertal level. The ICPP diagnosis and inclusion criteria were as follows: all patients showed a secondary sexual sign before eight years old, or menarche before ten years old; ovarian volume >1 ml; multiple follicles with diameter >4 mm; gonadotropin-releasing hormone (GnRH) stimulation test LH >5 IU/L and LH/FSH > 0.6. In addition, central tumor and injury were excluded by CT and MRI, as well as other organic diseases. All the healthy group children showed no prominent ICPP characteristics. Also, they did not use antibiotics and had no gastrointestinal symptoms such as diarrhea two weeks before fecal collection.

### 2.2. Fecal Sample Collection

Fresh stool samples from PPP, ICPP, and healthy children were collected using sample swabs (iClean, Shenzhen Huachenyang Technology Co., Ltd., China). The head of the swab was stored in sterile tubes (62-558-201, SARSTEDT AG & Co. KG, Germany) and transferred to −80°C for long-term storage within 30 minutes.

### 2.3. DNA Extraction and Analysis

All the bacterial DNA was extracted from fecal samples using the PowerSoil® DNA Isolation Kit (MO BIO, USA). The V3–V4 region of 16S rRNA gene was amplified and sequenced using Illumina Miseq. The 16S rDNA sequencing data were filtered and the paired-end reads were combined by Flash software (v1.2.11). Then the connected tags were clustered into OTUs (Operational Taxonomic Units) by USEARCH. The OTUs were annotated with the Greengene database (V201305), and their relative abundances were calculated. The differentially enriched bacteria among the three groups of ICPP, PPP, and HC groups were analyzed at the phylum, class, order, family, and genus levels.

### 2.4. GM Function Prediction and Symbiosis Network Construction

Based on 16S rDNA OTU analysis, PICRUSt obtained the function distribution of the gut microbiota under the default settings. The abundance of KEGG Orthology (KO) for each sample was calculated, and the enriched functional categories of the third and second levels of the KEGG database were detected.

### 2.5. Statistical Analysis

The ADE4 software package of *R* (v3.3.3) was used to analyze the compositions and relative abundances of the genus in all samples. Nonmetric multidimensional scaling analysis (NMDS) was carried out based on the profiling results, and the overall microbiota distribution of the three groups was exhibited. SPSS 23.0 was used for statistical analysis. Age, weight, and height of three groups were compared by two independent sample *t*-tests. *P* < 0.05 was considered to be statistically significant.

## 3. Results

### 3.1. Sample Characteristics and Data Output

In total, this study enrolled 27 ICPP children (ICPP group), 18 PPP children (PPP group), and 23 age-matched healthy children (HC group). The basic information, such as age, height, and weight were recorded and the weight and height of the participants in the PPP and ICPP groups were significantly higher than them in the HC group (*P* < 0.001, FDR < 0.001) (Table 1 and Supplementary Table 1). After the 16S rDNA sequencing of their fecal samples, the high-quality reads were connected into 2,194,015 tags, and the number of obtained OTUs was significantly higher in the PPP and ICPP groups when compared with the HC group (*P* < 0.001): the OTUs ranged from 181 to 458 in the PPP group, from 251 to 442 in the ICPP group, and from 62 to 221 in the HC group. After the annotation, we found that the number of identified genera was also significantly higher in the PPP and ICPP groups than that of the HC group (*P* < 0.001): the averaged genus numbers were 167, 223, and 125 in the PPP, ICPP, and HC groups (Supplementary Table 2).

### 3.2. The Differences of the GM Diversity among ICPP, PP, and HC Children

**Figure 1:**
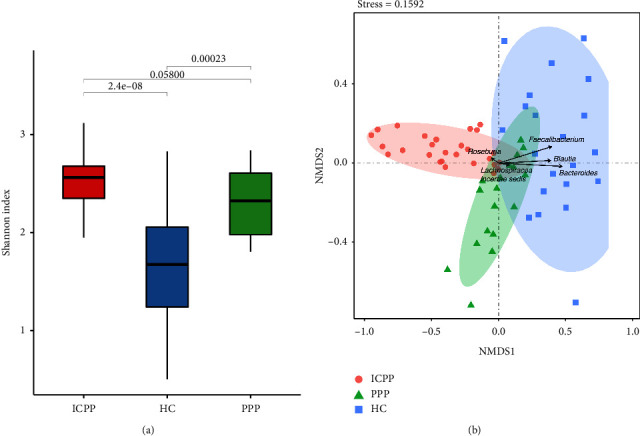
The microbiota diversity of the fecal samples from the PPP, ICPP, and HC groups. (a) The *α*-diversity of GM from the PPP, ICPP, and HC groups was shown by the Shannon index. (b) The NMDS analysis of all the samples from three groups and presented by red circle (ICPP), green triangle (PPP), and blue cube (HC).

To explore the *α*-diversity of the GM, Shannon index was calculated and the results illustrated that the GM *α*-diversity of the PPP group and the ICPP group was significantly higher than that of the HC group (*P* < 0.001) (Figure 1(a)), but there was no statistical difference in GM *α*-diversity between the PPP group and the ICPP group (*P* > 0.05). In addition, nonmetric multidimensional scaling analysis (NMDS) is used to explain the distribution and *β*-diversity of the samples within groups. The results presented that samples in three groups were scattered with a small area of overlap, but they were mainly clustered individually. The genera that contributed to the differences of three clusters were *Faecalibacterium*, *Bacteroides*, *Blautia*, *Roseburia,* and *Lachnospiracea incertae sedis* (Figure 1(b)).

### 3.3. Specific GM Differences among the Three Groups

**Figure 2:**
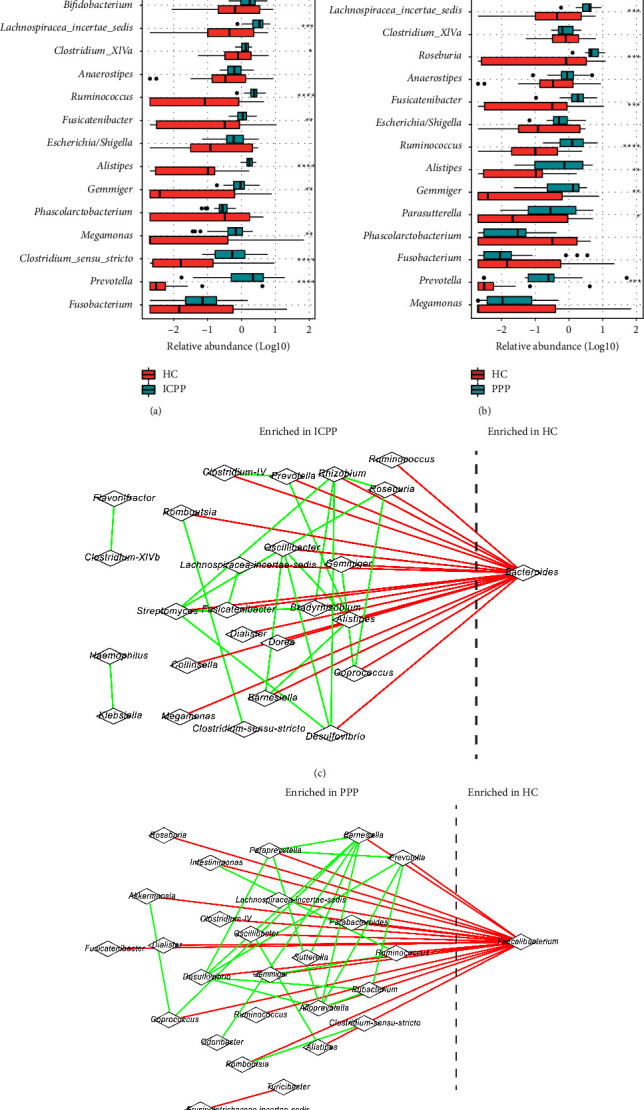
The differences of top abundant genera between the ICPP/PPP and HC groups, and the co-occurrence network of the genera. The abundances of genera were compared between the ICPP (a) or PPP (c) and HC group. The asterisks indicated the *P*-values. ^*∗*^, ^*∗∗*^, ^*∗∗∗*^, and ^*∗∗∗∗*^ stand for the *P*-value smaller than 0.05, 0.01, 0.001, and 0.0001, respectively. The genera enriched in the ICPP and HC groups (b), and the PPP and HC groups (d) were selected. Their co-occurrence networks were constructed. The green and red edges suggest the positive and negative correlations, respectively.

**Figure 3:**
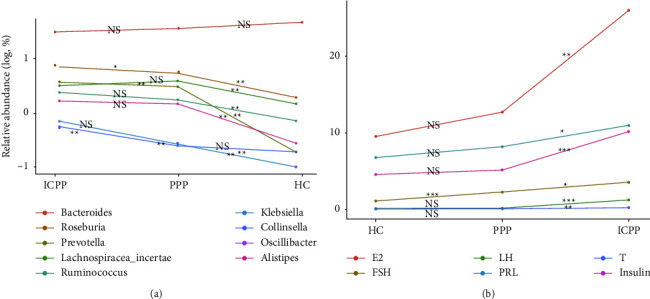
The abundance trend of the differential genera (a) and clinical phenotypes (b) in the HC, PPP, and ICPP groups. The asterisks and NS indicated the *P*-values. ^*∗*^, ^*∗∗*^, ^*∗∗∗*^, and NS (no significance) stand for the *P*-value smaller than 0.05, 0.01, 0.001, and *P*-value no less than 0.05, respectively.

Linear discriminant analysis (LDA) was applied to study the main genera that contributes to the GM differences among groups. After screening according to Kruskal–Wallis test *P* < 0.05, Wilcoxon test *P* < 0.05, and LDA > 2.0 with pairwise comparison between the three groups, the top 20 most abundant genera from HC, PPP, and ICPP were selected. Compared with the HC group, the PPP group and the ICPP group had significantly higher abundances of the following bacterial genera: *Prevotella, Lachnospiracea incertae sedis, Roseburia, Ruminococcus*, *Alistipes*, *Parabacteroides*, *Fusicatenibacter,* and *Gemmiger* (Figures 2(a) and 2(c)). The abundance of *Megamonas* in children in the ICPP groups was significantly higher than that in the HC group (*P* = 0.003, FDR = 0.006), but no significance was shown between the HC and PPP group. However, the abundance of *Bacteroides* in the ICPP group was significantly lower than that in the HC group (Figure 2(a)). In addition, *Faecalibacterium* showed a significantly lower abundance in the PPP group than the HC group (Figure 2(c)). In addition, all the significantly changed genera between the HC group and the ICPP/PPP group have also been selected and the GM networks were constructed, respectively (Figures 2(b) and 2(d)), which indicated that *Bacteroides* and *Faecalibacterium* were enriched in the HC groups when compared to the ICPP and PPP groups, respectively. Additionally, *Bacteroides* enriched in the HC group was negatively correlated with some beneficial bacteria that was enriched in the ICPP children, such as the butyrate-producing bacteria *Roseburia* and *Prevotella*. And in PPP children, *Faecalibacterium* was also negatively correlated with them. Furthermore, the difference of the abundance of genera presented among the three groups were also determined, and 9 genera showed significantly different (Figure 3). Interestingly, all of the genera showed a gradual increase from the HC group to the PPP group, then to the ICPP group, only except Bacteroides. This inferred that PPP might be an intermediate state between healthy and ICPP. In addition, the clinical phenotype, represented by the levels of estradiol (E2), follicle-stimulating hormone (FSH), luteinizing hormone (LH), prolactin (PRL), testosterone (T), and insulin, were determined. The result showed a continuous increasing trend from HC to PPP and to ICPP, which indicated that PPP maybe the intermediate clinical state of PP (Figure 3(a)). All of the hormone levels in PPP and ICPP were significantly higher than HC. Among them, FSH as a gonadotropin, whose elevation is one of the important indicators for the diagnosis of PP, showed significantly higher level in ICPP than in PPP, which may represent that ICPP is probably a more serious kind of PP.

### 3.4. GM Composition Correlated with Altered GM Functions

**Figure 4:**
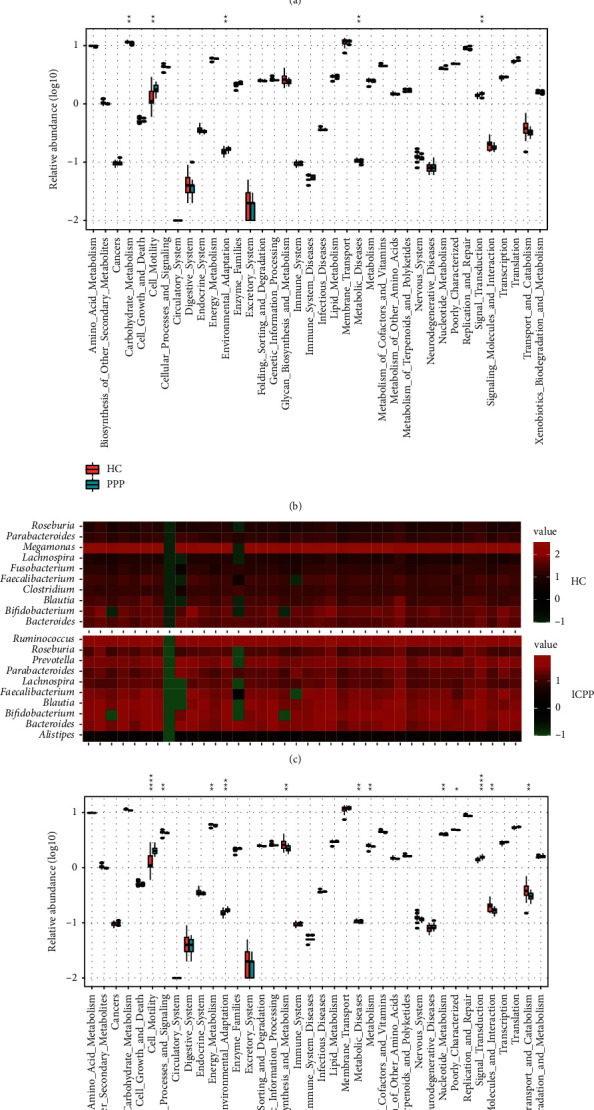
The distribution of the predicted GM function in the PPP and healthy groups. In the heatmap, the contributions of the top 10 genera on 38 KEGG functional categories were detected in the PPP and HC groups (a), and ICPP and HC groups (c), respectively. The deeper red square means the genera contribute to the functional category importantly, while the deeper green square means the functional category obtained less contribution from the genera. The enriched pathways were compared between the PPP and HC groups (b), and the ICPP and HC groups (d) and shown in the box plot. The asterisks indicated their *P*-values. ^*∗*^, ^*∗∗*^, ^*∗∗∗*^, and ^*∗∗∗∗*^ stand for the *P*-value smaller than 0.05, 0.01, 0.001, and 0.0001, respectively.

Applying PICRUSt and KEGG database, the function of the GM was predicted and 38 functional categories were obtained (Supplementary Table 3), 4 of which were significantly different between the groups of PPP and HC (*P* < 0.05), and 14 of which were significantly different between the groups of ICPP and HC (*P* < 0.05). The GM functional categories, including cell motility, signal transduction, and environmental adaptation were significantly enriched in the PPP group when compared to the HC group, while carbohydrate metabolism showed significantly higher relative abundance in the GM of the HC group (Figure 4(b)). Similarly, after the determination of the GM functional difference between the ICPP and HC groups, pathways related to cell motility, signal transduction, and environmental adaptation were also significantly enriched in the ICPP group, while carbohydrate metabolism pathway was also more abundant in the HC group (Figure 4(d)). In addition, 10 more pathways showed significant difference between the ICPP and HC groups, demonstration ICPP children had a more disordered GM. Moreover, all of the top 10 abundant genera contributed to the related GM metabolic pathways, which indicated that these functional differences were driven by the main compositions of the microbiome (Figures 4(a) and 4(c)).

## 4. Discussion

PP can affect children's physical and mental health by increasing the incidence of obesity, cardiovascular disease, and metabolic diseases, which is caused by complex factors [17, 18]. The gut microbiome has been confirmed to be one of the potential factors that associated with the occurrence of PP [13, 14]. In this study, for the first time, we looked into the relationship between GM and subgroups of the PP, including ICPP and PPP. We found that the GM composition and function of ICPP and PPP children is significantly different from health children. The alpha diversity of the gut microbiota in the PP (ICPP and PPP) children is significantly higher than that in the healthy children, which is consistent with the previous study [13, 14] (Supplement Table 4), while no significant change was observed between ICPP and PPP children. This indicated that the influence of the occurrence of disease on bacterial diversity was greater than the degree of disease. We also compared the pattern of gut microbiota in PP children and the children with obesity and nonalcoholic fatty liver disease, and PCOS patients (another disease closely related to hormone disorder) (Supplement Table 4). It seems that the relationship between GM and PP is more similar to its relationship with obesity.

Firstly, the abundance of some beneficial bacteria in ICPP and PPP children was declined. The current study found that the abundance of *Bacteroides* in the GM of children with ICPP significantly decreased, and *Bacteroides* was also less abundant in PPP children though no significance was shown. This result is consistent with the previous studies on the GM of obesity children (Supplementary Table 4), which is one of the common complications of PP children. Studies have confirmed that *Bacteroides* can degrade plant polysaccharides which cannot be digested by human body and provide 10%–15% of the energy from food [19]. The production of short-chain fatty acid, such as propionic acid, will be declined along the reduction of the abundance of *Bacteroides* [20], leading to the increase of ghrelin secretion [21], and further promoting the secretion of GnRH [22]. GnRH neurons send a long-distance projection to the median eminence and secretes GnRH in discrete pulses to the pituitary portal system to drive the release of gonadotropin, such as LH and FSH [23]. In this case, the frequency and quantity of pulsatile LH release will be increased and acted on gonads, leading to the development of sex signs, spermatogenesis, and follicular maturation, which resulted in precocious puberty. In addition, the decreasing trend of *Bacteroides* was shown from HC to PPP, and to ICPP, which corresponded with the development of PP. In addition, *Faecalibacterium* also presented a significant decrease in the PPP group than that in the HC group. *Faecalibacterium prausnitzii*, the sole known species belonging to *Faecalibacterium*, represents more than 5% abundant in the intestine of healthy people. *F. prausnitzii* is one of the key butyrate-producing bacteria in the intestinal tract. Butyrate provides energy and protects the intestinal mucosal barrier, and lack of butyrate can cause chronic inflammation of the intestinal mucosa [24]. The chronic inflammation can lead to nutritional attraction disorders and then induce metabolic diseases, such as obesity and diabetes [24], which is a common symptom appeared in precocious puberty children. Even the intestinal transplantation of *F. prausnitzii* was applied to prevent diabetes [25]. Therefore, the declined abundance of *Bacteroides* and *Faecalibacterium* would be a key factor of GM linking to the occurrence and development of PP.

Secondly, the abundance of some butyric acid-producing bacteria and conditional pathogenic bacteria in ICPP and PPP children increased. Compared with the HC group, *Prevotella, Lachnospiracea incertae sedis, Roseburia, Ruminococcus,* and *Alistipes* were enriched in the intestines of children in the PPP and ICPP groups, which was also shown in the previous studies [14]. Studies have confirmed that *Prevotella, Lachnospiracea incertae sedis,* and *Roseburia* can break down carbohydrates into short-chain fatty acids and participate in butyrate production [26–28]. Also, *Ruminococcus* is positively correlated with the butyric acid ratio in the large intestine [29]. In addition, *Alistipes* is closely related to high-sugar and high-fat diets [30, 31], and PP children prefer high-sugar and high-fat diets [32]. This result further confirms that changes in the diet of PP children affect the composition of GM. Furthermore, the abundance of *Alistipes* is closely related to the frequency of abdominal pain, which may cause intestinal inflammation [33]. Both butyrate and intestinal inflammation will stimulate insulin secretion and further enhance the transcription of GnRH gene by upregulating the mitogen-activated protein kinase (MAPK) pathway. The hypothalamus will respond to the increased expression of GnRH with increasing levels of androgens and LH secretion [34–36]. The increased abundance of *Alistipes* can secrete neurotransmission-related metabolites, such as acetic acid, serotonin, and dopamine, which activates the hypothalamic-pituitary-gonadal axis (HPGA) to trigger early puberty [37, 38].

The GM symbiosis networks constructed in this study showed the negative correlations between the bacteria (such as *Bacteroides* and *Faecalibacterium*) enriched in healthy children and bacteria enriched in ICPP/PPP patients (such as *Prevotella, Lachnospiracea incertae sedis, Roseburia, Ruminococcus,* and *Alistipes*). These antagonistic relationships indicated that the dominant growth of beneficial bacteria may inhibit the overgrowth of butyric acid-producing bacteria in mature GM individuals, and further keep the normal development of sexual maturity. Hence, we speculated that the abundance *Bacteroides*, *Faecalibacterium* and butyric acid-producing bacteria and the relationship between them may be the main contributor for the early puberty development.

The GM function was also predicted in this study, and the result manifested that the metabolic patterns of the GM were significantly different between the ICPP, PPP, and HC groups indicating that changes in the composition of GM result in the differences of function. Compared with the HC group, abnormal metabolic pathways related to cell motility, signal transduction, and environmental adaptation were all enriched in the ICPP and PPP children GM functions, while the high-sugar diets related pathway carbohydrate metabolism was reduced. The results remind that high-sugar intake should be limited in the clinical for PP children. Additionally, the identical significant change of the GM functional pathways mentioned above confirmed that these pathways played important roles in the development of PP, and may be the potential treatment targets.

## 5. Conclusions

Both ICPP and PPP children harbored excess butyric acid-producing bacteria in GM and lack of *Bacteroides* and *Faecalibacterium*, and microbial pathways related to carbohydrate metabolism declined. In addition, the changing trend of GM in healthy children during the transition to PP was clarified, which suggested that PPP may be a transitional stage between ICPP and HC children. However, limitations also existed in the current study, including lack of the large sample size and inaccurate function annotation based on 16S rDNA data. The finding in the current study is the first time the role of GM in the pathogenesis of different subgroups of PP is indicated, and provides a meaningful reference and theoretical basis for clinical grading intervention based on GM in the treatment of PP.

## Figures and Tables

**Table 1:** The comparison of the general information of the children in the three groups.

Anthropometrics	ICPP^1^ group (*n* = 27)	PPP^2^ group (*n* = 18)	HC^3^ group (*n* = 23)	*P*-value
Age (year)	7.72 ± 0.45	7.96 ± 0.53	7.4 ± 0.77	0.051
Height (cm)	138.89 ± 6.36	135.85 ± 8.69	129.87 ± 6.39	<0.001
Weight (kg)	34.92 ± 6.92	34.41 ± 8.16	26.31 ± 5.29	<0.001

^1^idiopathic central precocious puberty, ^2^peripheral precocious puberty, ^3^healthy children.

## Data Availability

The data set generated for this study can be read from the NCBI sequence Archive (SRA) database, biological project number: (PRJNA672248). This study was registered in China clinical trial center, this trial is registered with: ChiCTR2000033305.

## Supplementary Materials

Supplementary Materials Supplementary Table 1. The general information of each participant. Supplementary Table 2. The identified genera in the GM of each fecal samples. Supplementary Table 3. The detected 38 KEGG microbial functional categories of each fecal samples. Supplementary Table 4. The previous studies on the GM of obesity and nonalcoholic fatty liver disease children, and PCOS patient, which were the diseases all associated with PP.
